# Challenges and Solutions for Cost-Effective and Practical Dental Cast Fabrication With a Desktop Fused Deposition Modeling (FDM) 3D Printer

**DOI:** 10.7759/cureus.73354

**Published:** 2024-11-09

**Authors:** Takashi Kamio, Hiroshi Iwata, Taisuke Kawai

**Affiliations:** 1 Department of Oral and Maxillofacial Radiology, The Nippon Dental University, Tokyo, JPN; 2 Division of Oral Diagnosis, Oral and Maxillofacial Radiology and Pathology Diagnostic Services, The Nippon Dental University Hospital, Tokyo, JPN

**Keywords:** 3d printing, dentistry, fdm, patient-specific, simulation, thermal conductivity analysis

## Abstract

The objective of this study was to investigate the feasibility of using a cost-effective desktop three-dimensional (3D) fused deposition modeling (FDM) printer to fabricate dental casts to overcome the problems of conventional dental plaster casts, such as fragility and low portability.

First, a 3D computer-aided design (CAD) model of the dental cast was prepared in the Standard Triangle Language (STL) format. Twelve 3D models were fabricated using a desktop FDM 3D printer under different 3D printing parameters/conditions, including shape, placement direction, and infill percentage. The fabricated 3D models were reverse-scanned with a microfocus computed tomography unit. STL models were created from the scanned data and superimposed on a reference STL model to evaluate the effect of different parameters/conditions on the accuracy and quality of the 3D models. The results showed that the percentage of infill (25% vs. 75%) affected the accuracy and quality of the model. Thermal transfer simulations highlighted the role of internal structure/infill percentage in the deformation of the model during 3D printing.

In conclusion, although challenges such as thermal deformation and resolution limitations remain, it was found that even with an FDM 3D printer, the accuracy of 3D models can be improved by optimizing 3D printing parameters. This study demonstrates the feasibility of dental cast fabrication using an FDM 3D printer and may be one of the most cost-effective solutions. Depending on the future development of FDM technology, it is expected that this technology will be able to streamline the dental workflow and improve its efficiency.

## Introduction

Recent advances in technology have made it easier to create physical models from digital images. The adoption of a three-dimensional (3D) printing technology in dental practice, particularly in the preparation of dental casts and bite splints, has increased dramatically in recent years due to the widespread use of digital modalities such as intraoral optical scanners (IOSs) [1]. Dental casts serve various functions, including diagnostic applications and working casts in dental practice and dental laboratories. While conventional dental plaster casts have been around for a long time, they suffer from several drawbacks, such as single-use limitations, cumbersome weight, susceptibility to breakage during handling, and susceptibility to moisture and water, leading to problems during transportation and storage. In contrast, “digital” morphological data acquired with IOSs offer several advantages, including overcoming storage capacity constraints and facilitating information transfer to distant locations. As a result, there are high expectations for further use of such data. The use of 3D printers is expected to provide solutions to these challenges. The increasing familiarity with digital data suggests an impending trend of dentists increasingly using 3D printers to fabricate 3D models. In recent years, stereolithography 3D printers, which use ultraviolet lasers and projectors to solidify light-curing resin, have been widely used in dental laboratories because of their high accuracy. Meanwhile, oral and maxillofacial surgery is increasingly using osseous 3D models for purposes such as simulating osteotomies with practical surgical instruments and/or producing medical teaching materials [2]. For these applications, fused deposition modeling (FDM) systems, also known as fused filament fabrication (FFF) systems, are widely used [3]. The major advantage of FDM 3D printers is their affordability, which includes the 3D printer itself, the filament used as the 3D printing material, and the operational and maintenance costs. This economic viability positions FDM 3D printers as promising tools in dentistry and oral and maxillofacial surgery. However, there is a paucity of reports on the fabrication of dental casts using FDM 3D printers, and uncertainties remain regarding the suitability of these printers for applications beyond osseous 3D models. Addressing concerns related to the print and material properties of FDM 3D printers, such as their dependence on the placement direction, susceptibility to thermal deformation during printing, and comparatively lower definition compared to other 3D printing systems, is imperative to ensure the routine use of dental casts produced with FDM 3D printers [4,5]. This report highlights the challenges associated with fabricating dental casts with minimal deformation using a cost-effective approach on a desktop FDM 3D printer.

## Materials and methods

Definitions of terms

In this study, a 3D surface model (virtual 3D model) created in the Standard Triangle Language (STL) file format, which is a representative data format among the many types of 3D computer-aided design (CAD) data, is referred to as "STL data" or "STL model(s)." The dental cast fabricated from this STL data with a 3D printer is also referred to as "3D model(s)."

STL model creation

**Figure 1:**
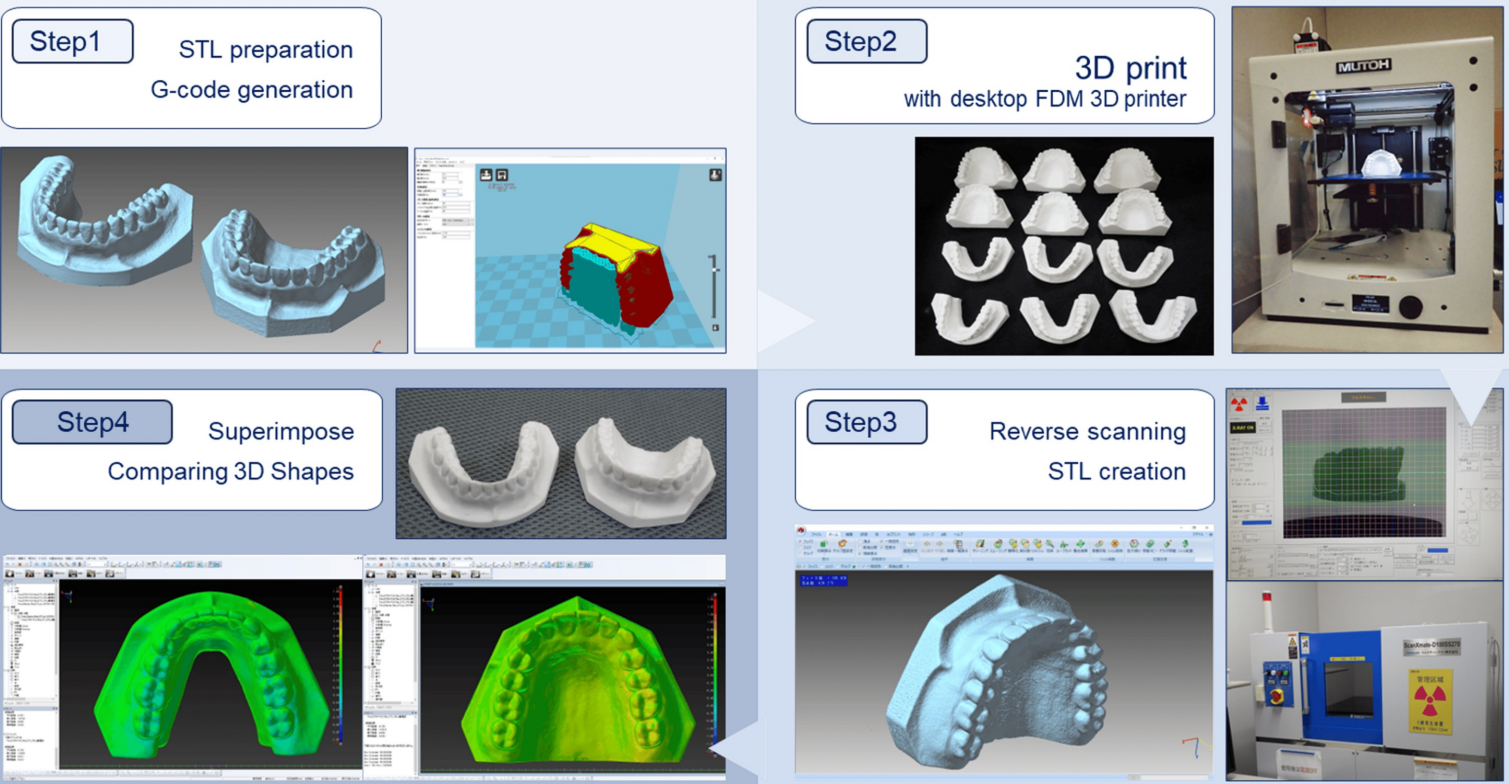
Process workflow from 3D printing of dental cast to shape error measurement STL: Standard Triangle Language; FDM: fused deposition model; CT: computed tomography Step 1: Preparation of STL model of dental casts and assignment of 3D printing parameters/conditions. Step 2: Fabrication with desktop FDM 3D printer. Step 3: Reverse scanning of a 3D model with microfocus X-ray CT and STL data creation. Step 4: Superimposition of STL models to visualize and measure shape errors. Image credit: Takashi Kamio, 2024

The workflow of the study is shown in Figure 1. In step 1, the general-purpose dental practice model was scanned with a dental cone-beam computed tomography unit (Finecube; Yoshida Dental Mfg Co. Ltd., Tokyo, Japan) with the following scanning parameters: 90 kV tube voltage, 4 mA tube current, 0.146 mm slice thickness, and 81 × 81 mm field of view. A 3D CAD model in STL format comprising approximately 250,000 polygons for both the maxilla and mandible was created and used as the reference/master STL model. A 3D image processing software was used for the creation and export of STL data from Digital Imaging and Communications in Medicine (DICOM) files. The size of the STL model created is 76.8 × 52.3 × 33.6 mm for the maxillary STL model and 77.3 × 34.2 × 52.6 mm for the mandibular STL model. In step 2, 12 3D models with different 3D printing parameters/conditions were fabricated on a desktop FDM 3D printer. In step 3, 12 fabricated 3D models were reverse-scanned with microfocus X-ray computed tomography (mCT). In this step, STL models for each model were created from the DICOM image data acquired on the mCT unit. In step 4, the reference STL model was compared to the respective STL models to evaluate shape errors.

3D printing parameters/conditions

**Figure 2:**
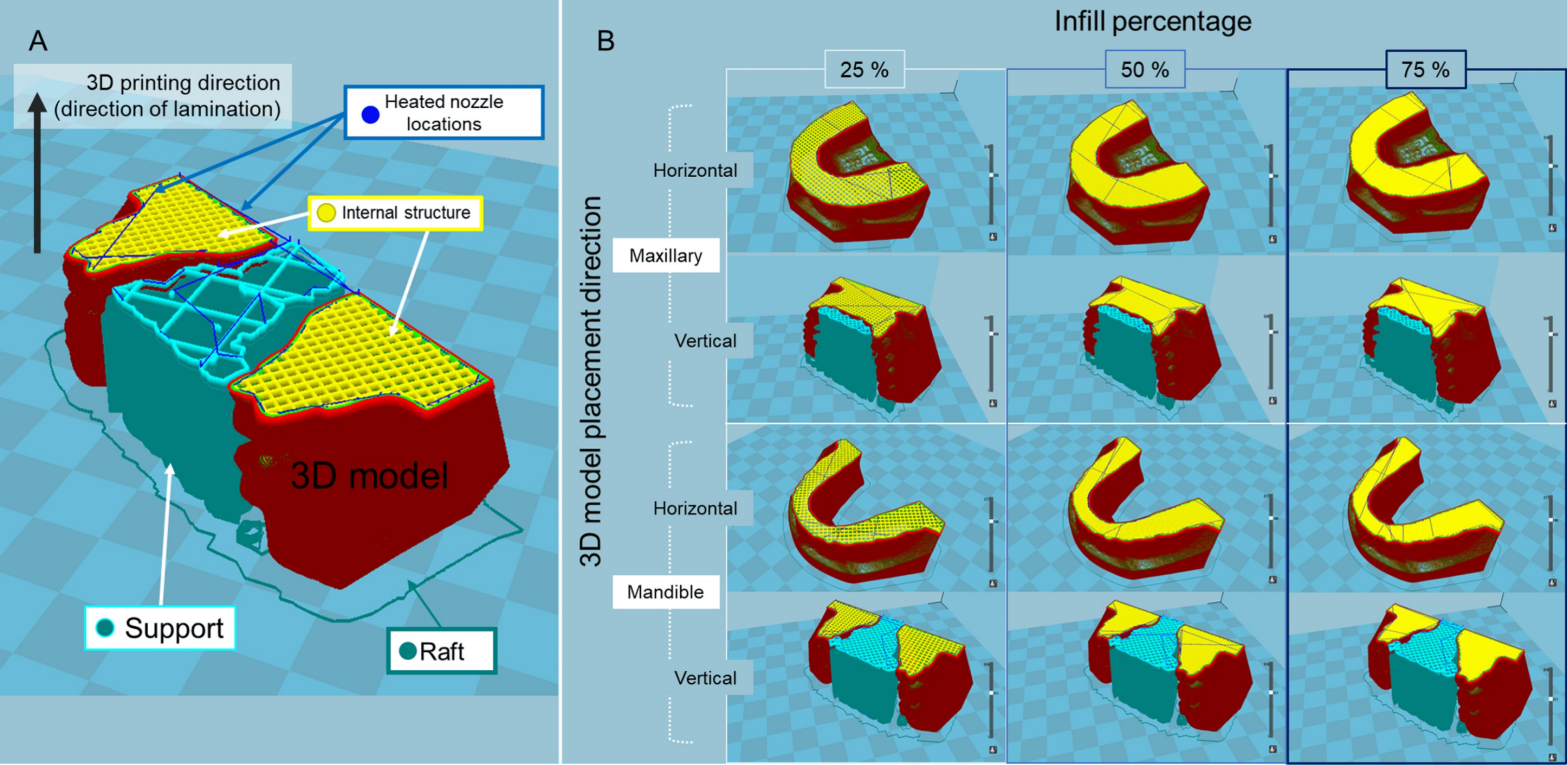
Overall images of 3D model shapes, 3D model placement directions, and infill percentages STL: Standard Triangle Language (A) Explanation of line colors in the figure. (B) Cross-section of the STL model for each 3D printing parameter/condition displayed in the slicing software. Image credit: Takashi Kamio, 2024

Twelve G-code files (mainly used in computer-aided manufacturing to control automated machine tools and in 3D printer slicer applications, where G stands for the geometry) were generated from the reference STL model data files using the slicing software. The respective parameters and conditions for 3D printing to be assigned to the G-code are presented in Figure 2 and Table 1.

**Table 1:** 3D printing parameters and conditions

Parameters and conditions	Value
Laminating pitch	0.1 mm
Print speed	50 mm/s
Flow rate	100%
Heated nozzle temperature	210 °C
Build plate temperature	60°C
With/without build plate adhesion	With raft at 3 mm extra margin
3D model placement direction-horizontal	Without support
3D model placement direction-vertical	With grid shape support at 20% infill density
Infill percentage	25%, 50%, 75%

The shape of the 3D model is approximately of trapezoidal shape with a palate for the maxilla and a horseshoe shape (U-shaped) for the mandible. The placement directions of the 3D models were horizontal to the basal plane of the 3D models (parallel to the occlusal plane) and vertical to the basal plane of the 3D models (perpendicular to the occlusal plane). The infill percentages (percentages of infill density) of the 3D models were 25%, 50%, and 75%.

Hardware and software

A desktop FDM 3D printer (Value 3D Magix MF-800; MUTOH Industries, Ltd., Tokyo, Japan) was used for 3D model fabrication, and a polylactic acid (PLA) filament (PolyLite PLA; Polymaker, Shanghai, China) that is a commercially available thermoplastic filament of 1.75 mm was used as the 3D printing material [6]. 3D printing parameters/conditions were assigned using slicing software (Cura 15.04; Ultimaker, Geldermalsen, The Netherlands). An mCT unit (ScanXmate-D300RSS270; ComscanTechno Co., Ltd., Kanagawa, Japan) was used for reverse scanning. Segmentation of DICOM images and creation of 3D CAD data in STL format after reverse scanning were performed using a 3D image processing software package (VolumeExtractor 3.0; i-Plants Systems, Iwate, Japan) [7]. In processing 3D CAD data, a software package (POLYGONALmeister version 9; UEL Co., Ltd., Tokyo, Japan) [8] was used for polygon editing. A 3D reverse engineering software (spGauge 2014.1, Armonicos Co., Ltd., Shizuoka, Japan) was used for the superimposition of STL models and evaluation of shape errors.

Thermal transfer simulation

The thermal transfer was simulated according to the hypothesis that the heat transfer from the build plate (also referred to as the heat bed or platform) of one of the 3D printer structures to the 3D model varies depending on the shape of the 3D model, the placement direction, and the infill percentages. A full-cloud computer-aided engineering simulation platform (SimScale; SimScale GmbH, Munich, Germany) [9] was used to analyze thermal transfer. Four datasets of STL models with different shapes, placement directions, and infill percentages were used to visualize the variations in heat transfer from the build plate to the 3D model. The density (ρ) was set as 1.17 g/cm^3^. The thermal conductivity was assumed to be isotropic with a constant thermal conductivity (k) of 0.13 W/(m･K) and a constant specific heat (C) of 1800 J/(kg･K).

Statistical analysis

Statistical analysis was performed using the statistical analysis language R version 4.1.2. A Mann-Whitney U test was conducted to compare the two groups, and a Kruskal-Wallis test was conducted to compare the three groups, with a value of *p* < 0.05 regarded as statistically significant.

## Results

Table 2 shows the 3D printing time and weight of each 3D model.

**Table 2:** 3D printing time and weight for each 3D model ^a^For both the maxilla and mandible, supports and rafts are provided if the placement direction of the 3D model is vertical. If the direction is horizontal, only rafts are provided. ^b^Regardless of the infill percentage of the 3D model, the characteristics of the provided support (configuration of the support and raft) are identical. ^c^3D printing time including supports and rafts. ^d^Weight after removal of supports and rafts

The shape of the 3D model ^a,b^	3D model placement direction ^a,b^	Infill percentage ^b ^ (%)	3D printing time^ c^	Weight of 3D model^ d ^ (g)
Maxillary	Horizontal	25	4 h 24 min	26
Maxillary	Horizontal	50	6 h 22 min	39
Maxillary	Horizontal	75	8 h 13 min	53
Maxillary	Vertical	25	6 h 22 min	28
Maxillary	Vertical	50	8 h 17 min	42
Maxillary	Vertical	75	10 h 18 min	58
Mandible	Horizontal	25	3 h 27 min	19
Mandible	Horizontal	50	4 h 47 min	28
Mandible	Horizontal	75	5 h 59 min	38
Mandible	Vertical	25	5 h 22 min	20
Mandible	Vertical	50	6 h 39 min	30
Mandible	Vertical	75	7 h 55 min	40

**Figure 3:**
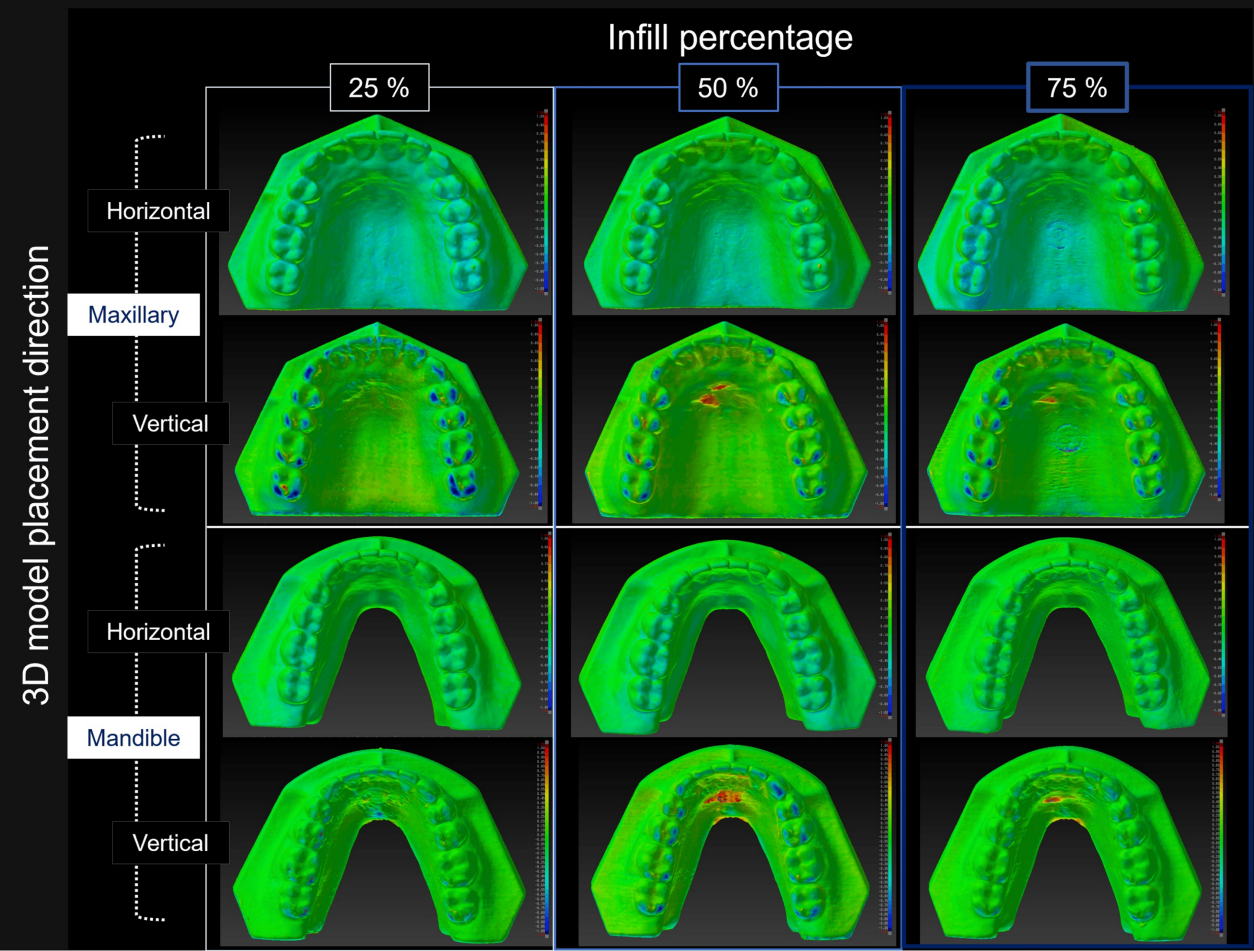
Visualization of the shape error (signed difference) for each STL model Warm colors indicate expansion from the reference STL model, while cold colors indicate shrinkage. In this color map, the maximum value for red represents 1.50 mm, and the minimum value for blue represents -1.50 mm. (A) Explanation of the surface colors in the figure. (B) Visualization of the shape error between the reference STL model and each STL model created by reverse scanning the 3D model. Image credit: Takashi Kamio, 2024

Figure 3 is a color map visualizing the geometric misalignment between the reference STL model and the STL model created by reverse scanning each 3D model.

Table 3 shows the mean, maximum, and minimum values of the shape error when comparing the STL model with the reference STL model. 

**Table 3:** Mean, maximum, and minimum shape error values between the STL model of the fabricated 3D models and the reference STL model STL: Standard Triangle Language

Shape	Placement direction	Infill percentage (%)	Mean (SD) (mm)	Maximum (mm)	Minimum (mm)
Maxillary	Horizontal	25	0.25 (0.30)	0.29	0.96
Maxillary	Horizontal	50	0.21 (0.29)	0.96	0.96
Maxillary	Horizontal	75	0.17 (0.22)	0.53	0.97
Maxillary	Vertical	25	0.23 (0.14)	0.92	0.49
Maxillary	Vertical	50	0.15 (0.21)	0.91	0.95
Maxillary	Vertical	75	0.10 (0.15)	0.46	0.46
Mandible	Horizontal	25	0.23 (0.31)	0.72	0.99
Mandible	Horizontal	50	0.17 (0.24)	0.54	0.96
Mandible	Horizontal	75	0.14 (0.15)	0.36	0.70
Mandible	Vertical	25	0.19 (0.27)	0.78	0.91
Mandible	Vertical	50	0.13 (0.15)	0.57	0.58
Mandible	Vertical	75	0.11 (0.15)	0.67	0.67

**Figure 4:**
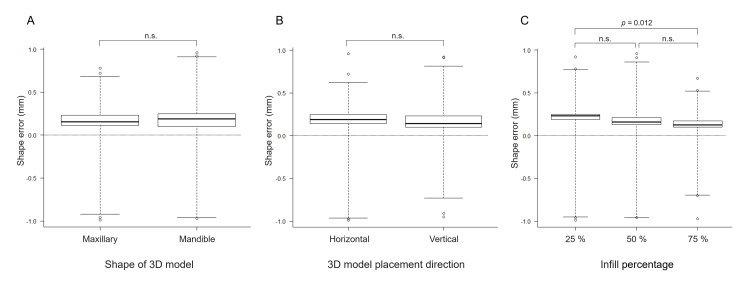
Shape errors between the reference STL model and the STL model created by reverse scanning each 3D model (A) 3D model shape, (B) 3D model placement direction, and (C) infill percentage. Statistical analysis showed significant differences only at 25% and 75% infill. Image credit: Takashi Kamio, 2024

The shape error (signed distance) between each STL model and the reference STL model was 0.17 ± 0.05 mm. Slight shape errors were observed on the surface of the 3D models with and without support. Still, there were no statistically significant differences between groups regarding the model shape (maxillary and mandibular) or direction of placement (horizontal or vertical) of the 3D model. Meanwhile, statistically significant differences in the infill percentages were observed between the groups (25% vs. 75%) (Figure 4).

## Discussion

The results of 3D printing with different 3D printing parameters and conditions showed that as the infill percentage increased, the 3D printing time and the weight of the 3D model also increased. The 3D models with 25% infill tended to have larger average shape errors. However, the errors that occurred were numerically negligible. The clinically acceptable shape error varies depending on the application of the 3D model. Therefore, this study will consider "the accuracy with which occlusal conditions can be confirmed."

**Figure 5:**
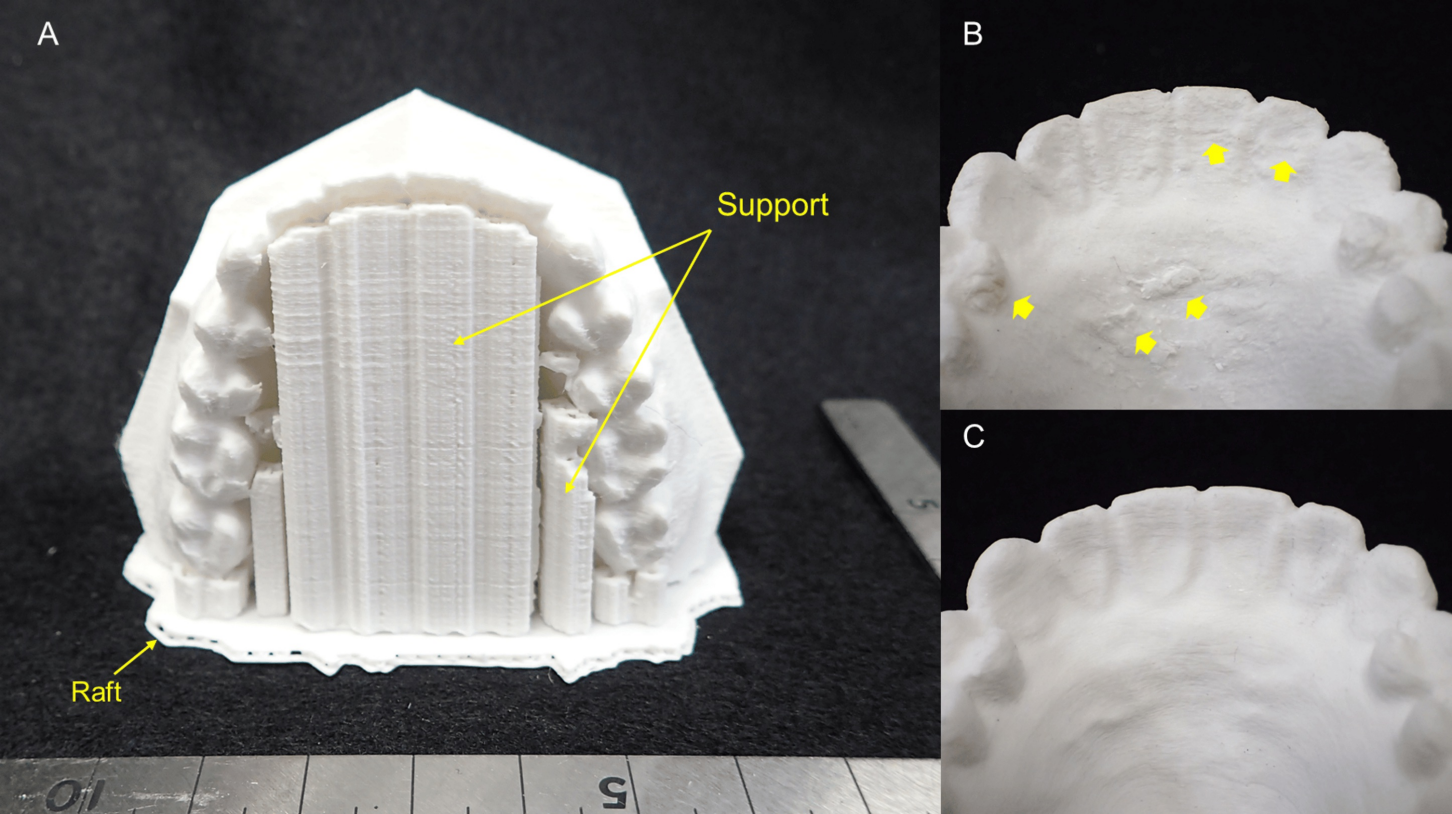
Examples of 3D models fabricated on a desktop FDM 3D printer (A) Overview of support and raft placement. (B) Shape: maxillary; 3D model placement direction: vertical; infill percentage: 50%. The rough surface of the 3D model is due to the difficulty of removing the tightly bonded supports (arrows). (C) Shape: maxillary; 3D model placement direction: horizontal; infill percentage: 50%. Since no support was provided, the surface of the 3D model is smooth

The global boom in 3D printing in the 2010s has resulted in smaller and less expensive digital machine tools, making it easier to bring the entire process, from design to manufacturing, in-house. This trend is seen in dentistry, where the generalization of 3D printers has enabled a series of processes to be carried out in the dental clinic. This has solved the distance and time problems of exchanging dental casts to and from dental laboratories. The accelerated development of low-cost desktop 3D printers and their widespread use is thought to have accelerated this trend [10]. The desktop FDM 3D printer and polylactic acid (PLA) filament used in this study are by no means unique and are universally available products. The price of the 3D printer is about $1500, and the filament is available for about $30 per 1000 grams. A PLA was selected as the 3D printing material in this study as PLA is a typical biodegradable plastic produced from plant-derived renewable resources and has attracted attention for its petroleum-independent and soil-returning properties against a background of growing environmental awareness [11]. PLA has many advantages in FDM 3D printing, including dimensional stability (less deformation), 3D printing stability (less nozzle clogging), and low cost. It was selected because it is currently one of the leading filaments for FDM 3D printers and is used in a wide range of applications [12]. This study aimed to investigate whether a desktop FDM 3D printer could fabricate 3D models with practical accuracy in oral and maxillofacial surgery, particularly in the treatment of jaw deformities requiring collaboration between the orthodontist and the oral and maxillofacial surgeon. It is no exaggeration to say that one of the most important events in the long-term treatment of jaw deformities is the detailed consultation between the oral and maxillofacial surgeon performing the surgery and the orthodontist responsible for the occlusion before surgery. In their discussion, the surgeon and orthodontist develop an understanding and consensus on the final surgical plan using, for example, a dental cast mounted on an articulator that presents the direction and amount of maxillary and mandibular movement and the limits of that movement. Therefore, both are encouraged to be present for the discussion [13-15]. With the combination of digital data and a 3D printer, it is possible to easily duplicate multiple dental casts. In the future, the ability to fabricate dental casts on a 3D printer in one's own office will allow this type of discussion to take place even when the attending physician is in a remote location which makes it difficult to meet. In this study, the shape errors were visualized on a color map by superimposing the reference STL model and the STL model of the fabricated 3D models. Opinions may differ as to whether this result should be considered "high precision" or "low precision." Obviously, given the minimum lamination pitch (0.1 mm) of FDM 3D printers, it is too coarse for dental lab applications that require precision of 0.1 mm or less. However, the 3D models that were fabricated did not show any noticeable difference from plaster dental casts, and I felt that they were "light and strong." The results show slight partial differences between the STL data, and the average and maximum confirmed dimensional errors were 0.17 and 0.24 mm, respectively. The color map shows that all 3D models are slightly expanded from the original design data. However, the results of the statistical analysis show no significant differences in the 3D printing parameters/conditions except for the 25% and 75% infill percentages. Two possible causes of shape errors in the fabrication of 3D models from the same STL data are (1) the deformation of the 3D models during 3D printing and (2) the difficulty in removing the supports required for 3D printing. Indeed, some 3D models with supports attached were difficult to remove. As a result, the surfaces of the 3D models became rough (Figure 5).

**Figure 6:**
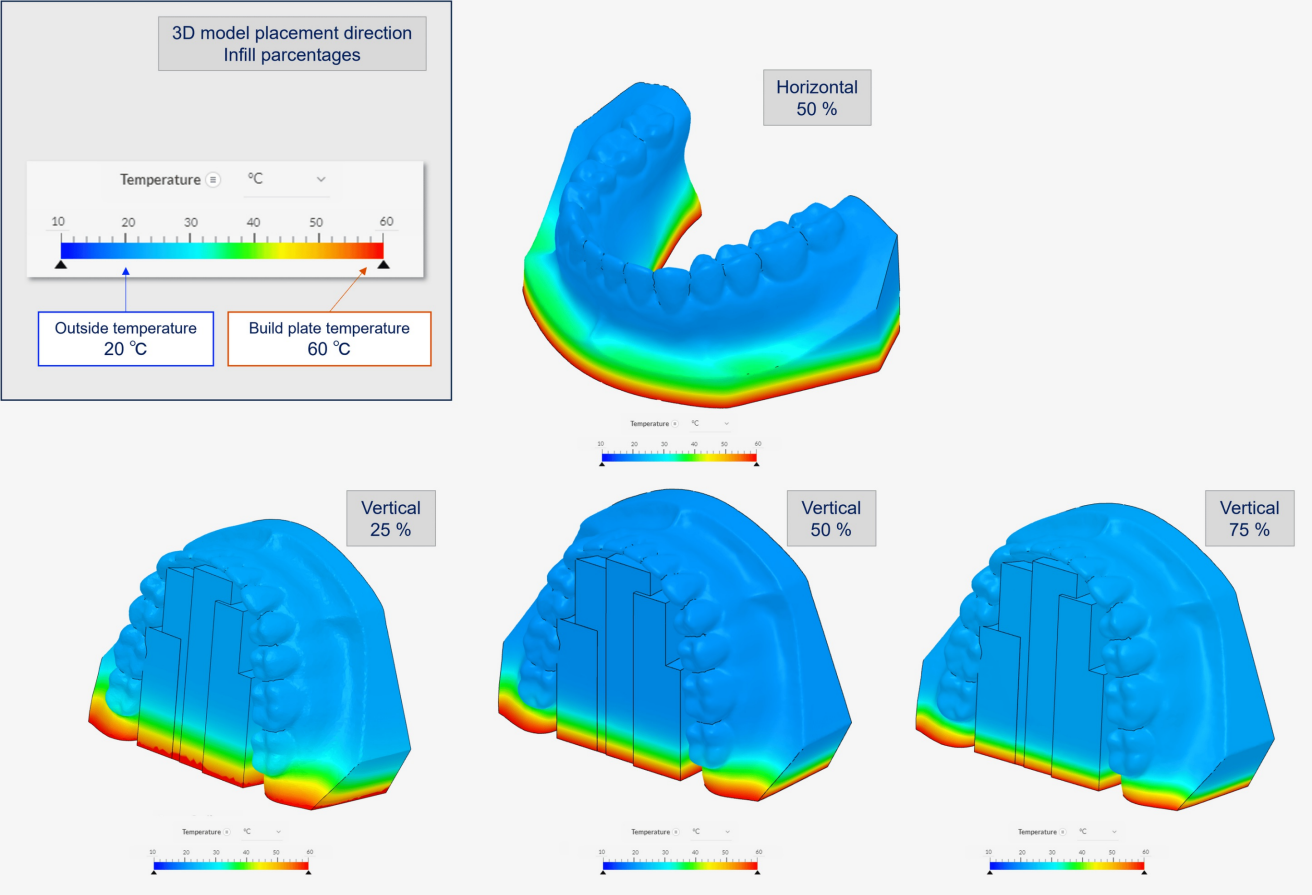
Thermal transfer simulation with different 3D model placement direction and infill percentage using SimScale STL: Standard Triangle Language In the upper left box of the figure, the top row shows the placement direction of the 3D model, and the bottom row shows the infill percentage. The outside temperature is set to 20℃, and the bottom temperature is set to 60℃, the build table temperature. Before the simulation, the raw STL model's polygon count was reduced using POLYGONALmeister because it was too large to calculate. Image credit: Takashi Kamio, 2024

The results suggest the importance of considering the parameters/conditions under which support is provided, e.g., the positions of supports, clearance between the 3D model and supports, shapes, and infill percentages of the supports. PLA has a low glass transition temperature (approximately 60℃-65℃) and tends to soften and expand under high temperatures while shrinking and returning to its original shape when cooled [16,17]. Therefore, it is not difficult to imagine that the shape of a 3D model may be affected by the temperature setting during 3D printing. The thermal transfer simulations were thus performed to confirm the heat-induced deformation, with the results shown in Figure 6.

The heat transfer from the 3D printer build plate to the 3D model was found to vary depending on the shape, placement direction, and infill percentages of the 3D model. Based on these results, it was inferred that the internal structure of the 3D model, the infill percentage, and the temperature imbalance from the bottom to the top of the 3D model were also contributing factors to the deformation of the 3D model.

The author has previously reported that desktop FDM 3D printer is easy to use (the 3D printing process starts as soon as the 3D printer loads the STL file); however, it inevitably takes a lot of time and effort to become proficient in its use and to be able to deal with the various 3D printing problems that arise during use [3]. Furthermore, there are limitations in terms of fabrication accuracy. According to the manufacturer's official specifications, the minimum lamination pitch is 0.1 mm, which is the same resolution as many other desktop FDM 3D printers. It is of course not suitable for fabricating 3D models such as dental prostheses, which require high accuracy of 0.1 mm or less. There have been many reports on the use of 3D printing technology to produce 3D models for dental applications and to validate their accuracy. Rungrojwittayakul et al. [18] reported on a comparison of the accuracy of typodont models produced using two 3D printing technologies, a Continuous Liquid Interface Production (CLIP) 3D printer and a Digital Light Processing (DLP) 3D printer. They reported that the shape error between the 3D models produced by these 3D printers and the reference data was less than 100 μm for both. The results of this study using FDM 3D printing technology showed a shape error of 170 mm. We conclude, as they did, that clinically acceptable levels of accuracy can be achieved even with low-cost desktop FDM 3D printer. To create the reference STL model for this study, the standard dental practice model was scanned with a CBCT. In terms of data acquisition, the IOSs and optical scanners for dental laboratories are considered superior in terms of spatial resolution. However, the ease of creating 3D CAD data with CBCT suggests that CBCT can be used as a tool for duplicating dental models for applications such as those described in this study. Dental casts fabricated using FDM 3D printers have features that plaster dental casts do not have, such as impact resistance, fracture resistance, and ease of transportation. However, 3D models fabricated with general-purpose PLA resin filaments, such as those used in this study, cannot withstand the operation temperatures of dental thermoforming machines (generally 200℃ or higher) used in the fabrication of orthodontic appliances and mouthpieces, resulting in poor resistance to compression and easy deformation. Currently, no high-temperature heat-resistant general-purpose filament is commercially available, making PLA resin filaments unsuitable for these applications.

The results of this study demonstrate the importance of 3D CAD data design and setting fabrication parameters/conditions, considering the placement of supports that make heat transfer to the 3D model as uniform as possible and minimize the effect on the model surface profile. It is not difficult to imagine that 3D printing technology will continue to evolve and become more widespread, given the recent remarkable progress in the use of "digital" morphological data with IOSs. On-demand and speedy fabrication of 3D models in dental offices would solve the problem of storage. These 3D models will be a realistic communication tool with distant physicians because they can be touched, rather than viewed on a two-dimensional (2D) computer screen. In addition, low-cost 3D models can be used for various training programs in medical education without the need to purchase expensive ready-made models. As of 2024, desktop FDM 3D printers will have the disadvantage of taking a long time to fabricate 3D models, but technological advances will solve this challenge soon.

## Conclusions

In conclusion, the challenges of fabricating 3D models of dental casts using a desktop FDM 3D printer were addressed. Parameters such as placement direction, infill percentages, and thermal deformation that affect the accuracy of the model were investigated. The results showed that desktop FDM printer, while affordable, requires optimization of fabrication parameters/conditions to minimize shape errors. There are great expectations for improving the efficiency of dental practices, facilitating collaboration in remote areas, and enhancing medical education. Advances in digital and 3D printing technologies are expected to further streamline workflow and improve patient care in dentistry.

## References

[1] Mangano F, Gandolfi A, Luongo G, Logozzo S (2017). Intraoral scanners in dentistry: a review of the current literature. BMC Oral Health.

[2] Narita M, Takaki T, Shibahara T, Iwamoto M, Yakushiji T, Kamio T (2020). Utilization of desktop 3D printer-fabricated "Cost-Effective" 3D models in orthognathic surgery. Maxillofac Plast Reconstr Surg.

[3] Kamio T, Onda T (2022). Fused deposition modeling 3D printing in oral and maxillofacial surgery: problems and solutions. Cureus.

[4] Taczała J, Czepułkowska W, Konieczny B (2020). Comparison of 3D printing MJP and FDM technology in dentistry. Arch Mater Sci Eng.

[5] Quan Z, Suhr J, Yu J (2018). Printing direction dependence of mechanical behavior of additively manufactured 3D preforms and composites. Compos Struct.

[6] (2024). Polymaker technical data sheet: Polylite TM PLA. https://www.poly-maker.jp/download/TDS/PolyLite_PLA_TDS_V5.1.pdf.

[7] Doi A, Takahashi T, Mawatari T (2012). Development of volume rendering system using 3D texture display techniques and its applications. Med Imag Tech.

[8] Tanimoto S (2012). Collaborative research on polygon engineering with RIKEN (Article in Japanese). Unisys Technol Rev.

[9] (2024). SimScale: a cloud-based simulation platform. https://www.simscale.com/.

[10] Azari A, Nikzad S (2009). The evolution of rapid prototyping in dentistry: a review. Rapid Prototyp J.

[11] Chen H, Yang X, Chen L, Wang Y, Sun Y (2016). Application of FDM three-dimensional printing technology in the digital manufacture of custom edentulous mandible trays. Sci Rep.

[12] Pang X, Zhuang X, Tang Z, Chen X (2010). Polylactic acid (PLA): research, development and industrialization. Biotechnol J.

[13] Steed MB, Crisp HA, Perciaccante VJ, Bays RA (2022). Model surgery and computer-aided surgical simulation for orthognathic surgery. Peterson’s Principles of Oral and Maxillofacial Surgery.

[14] Yosano A, Yamamoto M, Shouno T (2005). Model surgery technique for Le Fort I osteotomy-alteration in occlusal plane associated with upward transposition of posterior maxilla. Bull Tokyo Dent Coll.

[15] Labib A, Mohamed AF, Allouba K (2008). Identification of posterior nasal spine for assessment of horizontal maxillary rotation. Egypt Orthod J.

[16] Lanzotti A, Grasso M, Staiano G (2015). The impact of process parameters on mechanical properties of parts fabricated in PLA with an open-source 3-D printer. Rapid Prototyp J.

[17] Gonabadi H, Yadav A, Bull SJ (2020). The effect of processing parameters on the mechanical characteristics of PLA produced by a 3D FFF printer. Int J Adv Manuf Tech.

[18] Rungrojwittayakul O, Kan JY, Shiozaki K, Swamidass RS, Goodacre BJ, Goodacre CJ, Lozada JL (2020). Accuracy of 3D printed models created by two technologies of printers with different designs of model base. J Prosthodont.

